# Competitive Interactions between Invasive Nile Tilapia and Native Fish: The Potential for Altered Trophic Exchange and Modification of Food Webs

**DOI:** 10.1371/journal.pone.0014395

**Published:** 2010-12-21

**Authors:** Charles W. Martin, Marla M. Valentine, John F. Valentine

**Affiliations:** 1 Dauphin Island Sea Lab, Dauphin Island, Alabama, United States of America; 2 Department of Marine Science, University of South Alabama, Mobile, Alabama, United States of America; California Academy of Sciences, United States of America

## Abstract

Recent studies have highlighted both the positive and negative impacts of species invasions. Most of these studies have been conducted on either immobile invasive plants or sessile fauna found at the base of food webs. Fewer studies have examined the impacts of vagile invasive consumers on native competitors. This is an issue of some importance given the controlling influence that consumers have on lower order plants and animals. Here, we present results of laboratory experiments designed to assess the impacts of unintended aquaculture releases of the Nile tilapia (*Oreochromis niloticus*), in estuaries of the Gulf of Mexico, on the functionally similar redspotted sunfish (*Lepomis miniatus*). Laboratory choice tests showed that tilapia prefer the same structured habitat that native sunfish prefer. In subsequent interspecific competition experiments, agonistic tilapia displaced sunfish from their preferred structured habitats. When a piscivore (largemouth bass) was present in the tank with both species, the survival of sunfish decreased. Based on these findings, if left unchecked, we predict that the proliferation of tilapia (and perhaps other aggressive aquaculture fishes) will have important detrimental effects on the structure of native food webs in shallow, structured coastal habitats. While it is likely that the impacts of higher trophic level invasive competitors will vary among species, these results show that consequences of unintended releases of invasive higher order consumers can be important.

## Introduction

Although debated recently [1]–[4], it has historically been accepted that successful biological invasions detrimentally affect the structure and function of native ecosystems [5]–[7]. In fact, according to the National Research Council [8], biological invasions represent “one of the five most critical environmental issues facing the ocean's marine life.” Recent articles of invasive plant impacts on native plant species richness, however, do not always lend support to this paradigm [9]–[11]. What impacts higher order invasive species have less are certain, as fewer studies are available to test the validity of these beliefs [2], [11]. Even so, it is reasonable to predict their impacts would be intense, given the controlling role that such consumers can have in structuring ecosystems [12]–[17].

The rising numbers of invasive species in marine and estuarine waters are thought to be due to the ever increasing human migration to the world's coastlines [18], transport of organisms across geographic dispersal barriers [19], and further urbanization of coastal ecosystems [6]. Concurrent with these perturbations is the probable creation of vacant niches following depletion of native marine fishes by overfishing [20]–[21].

Among the solutions proposed to lessen fishing pressure on coastal resources has been the increased the use of aquaculture [22]–[24]. Poorly managed aquaculture can, however, have deleterious impacts on the environment [25], including increasing incidences of: 1) eutrophication [26]–[27], 2) disease/parasitism in native species [28]–[30], 3) accidental releases of non-native aquaculture organisms into surrounding waters [31], and 4) alterations of vital coastal ecosystems [32]–[33]. Despite these risks, aquaculture is widely used by many nations to increase food production [25].

Among the most popular of the fishes used in aquaculture is the Nile tilapia (*Oreochromis niloticus*). Nile tilapia are members of the Family Cichlidae, whose members have successfully invaded ecosystems worldwide [34]–[36]. Many of the characteristics that make tilapia desirable also allow them to proliferate in areas outside their native range [37]–[40]. Tilapias are tolerant of wide fluctuations in salinity, dissolved oxygen, and temperature [41]–[44]. This tolerance to environmental variability, along with their high fecundity [45], rapid growth rates [46]–[47], and omnivorous feeding habits [48] further contribute to successful invasions in estuaries.

Published and anecdotal reports both indicate that tilapia have successfully colonized oligohaline habitats in many areas of the northern Gulf of Mexico (NGOM) (including Florida [49]–[56], Alabama (anecdotal collections), Mississippi [39]–[40], [57]–[59], Louisiana [60], and Texas [54], [61]). Although tilapia are reported to perish at temperatures <10°C [62], tilapia can find thermal refuges (e.g., deeper waters and warm industrial thermal plumes) that allow them to survive episodically cold winters in the northern gulf [59], [63]. With the predicted rises in temperature associated with global climate change, and the warmer winters recently observed in the area [64], it is reasonable to hypothesize that tilapia now persists in many areas of the northern gulf. The impacts of the release of most aquaculture species on native fishes remain unknown. Of the studies that have been done, most are descriptive and are focused on comparisons of dietary overlap with native fishes [e.g., [39]–[40],[57]–[59]]. Indirect community impacts of agonistic tilapia, however, have yet to be documented.

The repeated reports of tilapia being present in the NGOM is alarming because the oligohaline reaches of these areas are considered to be hot spots of biodiversity that contain a diverse mix of fresh and saltwater species [65]. In coastal Alabama, for example, more than 150 species of fish use the watershed as nursery grounds [66], including a number of commercially and recreationally important estuarine species such as spotted seatrout, flounder, red drum, mullet, brown shrimp, and blue crabs, as well as freshwater species such as largemouth bass, blue and channel catfish, and several species of Centrarchid sunfish. It is possible therefore, that the impacts of tilapia may have been catastrophic for native biodiversity, especially if their invasion resulted in the competitive exclusion of native species from protection of structured habitats as would be hypothesized based on their aggressive nature.

Here, we describe the results of a series of experiments designed to assess: 1) the extent to which unintended releases of tilapia have altered the habitat utilization patterns of one abundant native fish (the redspotted sunfish *Lepomis miniatus*) and 2) determine if there are consequences for *L. miniatus* survival if they are inferior competitors for a mutually preferred habitat.

## Methods

### Experimental Organisms

To identify tilapia's habitat preferences and to evaluate their impacts on the habitat preferences of native fishes in coastal ecosystems, we elected to use one of the most abundant species of native sunfish found in the oligohaline habitats of coastal Alabama, the redspotted sunfish (*Lepomis miniatus*). Based on the salinity tolerance (which reaches 20 psu) and distributional maps of *L. miniatus* [66], as well as the reported locations of tilapia, it is likely that these species co-occur in many estuaries throughout the NGOM. We selected a similarly abundant predator in these same estuaries, the largemouth bass (*Micropterus salmoides*) for use in predator-prey experiments. Both sunfish and bass were collected in the Mobile-Tensaw Delta using a 6 m otter trawl. Tilapia used in these experiments were donated by Gadsden State Community College Aquaculture Education and Development Center. This study was reviewed by the University of South Alabama Department of Marine Science and Dauphin Island Sea Lab and approval was received via the issuance of permits to collect by the state of Alabama Department of Conservation and Natural Resources (Permit #: 2010000052468680 NH30501570251O24).

### Experiment 1: Competitive Exclusion

To determine if tilapia can competitively exclude redspotted sunfish from their preferred habitat, we performed choice experiments in 98L tanks located in the Dauphin Island Sea Lab's (DISL) recirculating wet lab facility to prevent release of tilapia into the adjacent waterways and also because poor visibility and the heterogeneous distribution of vegetated habitats hindered proper identification of behavioral interactions in a field setting. Tanks contained equal areal coverages of either bare sediment or artificial submerged aquatic vegetation (SAV), constructed of equal length green ribbon at 100 stems m^−2^, similar in appearance to, and within the range of densities recorded for, *Vallisneria americana*, the dominant native species of submerged aquatic vegetation in many NGOM estuaries. The ribbon was tied onto plastic Vexar (DuPont®) mesh, which was buried in the sediment [see 67]. Salinity, held constant at 5psu, paralleled measurements made at sites where the bass and sunfish were collected and where tilapia is known to occur in the region [39]. Artificial lighting, on a 12 h light∶dark cycle, was used to approximate natural light cycles. All fish were held in separate tanks until used in experiments. No organism was used twice in trials. The mean sizes (total length) of species (tilapia: 7.3±0.48 mm; sunfish: 7.29±0.50 mm) paired in the trials were statistically indistinguishable from each other (t_66_ = −0.035, p = 0.973).

In this experiment, treatments consisted of three combinations of the two species: two tilapia singly, two sunfish singly, or one sunfish and one tilapia. In single species trials, two sunfish or two tilapia were used to document the habitat preference patterns of each fish in the absence of the other. Sunfish density was within the natural range of densities found in the area [1.68±0.68 m^−2^; 68]. At the beginning of each trial, one of the aforementioned fish treatments was randomly selected, then the fishes in the holding tanks were transferred to the center of each tank. Fish movements between habitats were documented for 1 h using a Sony digital video camera. Video recordings were analyzed and the proportion of time each fish spent in the habitats (the artificial structure or bare sediment) was recorded. In analyzing trials with two fish of the same species, the movements of one randomly selected fish was followed throughout the experimental period. A one-way ANOVA was used to compare the proportion of time spent in structured habitat (arcsine square transformed) among the three treatment combinations (2 tilapia, 2 sunfish, 1 sunfish+1 tilapia) after assumptions of the tests (normality and homogeneity of variance) were satisfied. Statistics were performed using SPSS v16.0. Arcsine square transformations were performed on proportion data and the results considered significant at p≤0.05.

### Experiment 2: Impacts on Native Sunfish Survival

To determine if a significant shift in habitat use by the sunfish occurred in the presence of tilapia, and if there was a consequence should a shift occur, we used a larger tank (492 L) to accommodate the presence of multiple prey as well as a large predator. The same artificial lighting regime was used to mimic field conditions as described above and no fish was used twice in trials.

In these trials, a patch (0.40cm×0.40cm) of artificial structure (100 stems m^−2^), similar in construction to that used in Experiment 1, was randomly placed in the tank. Five tilapia and five sunfish were released into the center of the tank and allowed to acclimate to laboratory conditions for 30 min, then the predator was released into the tank. After 1 h, the bass was removed and number of survivors of each species was recorded. Mean bass sizes (total length) were consistent among trials (266±13.5 mm).

Separate two-tailed, one sample, t-tests were used to compare survivorship (arcsin square root transformed) of redspotted sunfish and tilapia in trials with and without artificial structure. The response variable (survivorship difference) for each test was calculated following:
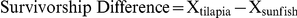
Where X_tilapia_ refers to the proportion of tilapia surviving and X_sunfish_ refers to the proportion of sunfish surviving at the end of each trial. The survivorship difference served as the response variable to determine if the mean varied significantly from zero [c.f., 69]. This was done to avoid pseudoreplication (e.g., if the largemouth bass eats a sunfish then it cannot theoretically eat a tilapia at the same time, thus the two survival percentages are not independent) thus making the test more conservative. Assumptions of the tests were checked using Kolmogorov-Smirnov (normality) and Bartlett's χ^2^ test (homogeneity of variance). Statistics were performed using SPSS v16.0 and arcsine square root transformations performed on proportion data and results considered significant at p≤0.05.

## Results

### Experiment 1: Competitive Exclusion

**Figure 1:**
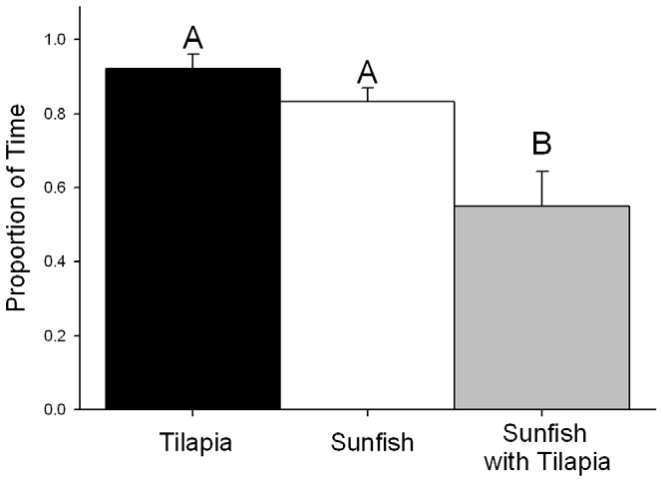
Proportion of time (s; mean ±1 standard error) spent in structured habitat during lab trials for each species treatment. Differences in upper case letters indicate significant differences between treatments (p≤0.05).

The amount of time each species spent in structured habitat varied significantly among treatments (Figure 1; F(2,21) = 10.82, p = 0.001). Data satisfied assumptions of normality (D = 0.199, p = 0.267) and homogeneity of variance (χ^2^ = 2.620, p = 0.270). Both Nile tilapia and sunfish occupied the structured habitat significantly more often than they did the sand habitat in single species treatments (Figure 1). However, when both species were present, the amount of time that sunfish spent in the structured habitat was significantly lower than in either monoculture trials (sunfish: p = 0.014; tilapia: p = 0.001). See supplemental online video (Video S1) for documented examples of aggressive interactions between tilapia and sunfish.

### Experiment 2: Impacts on Native Sunfish Survival

**Figure 2:**
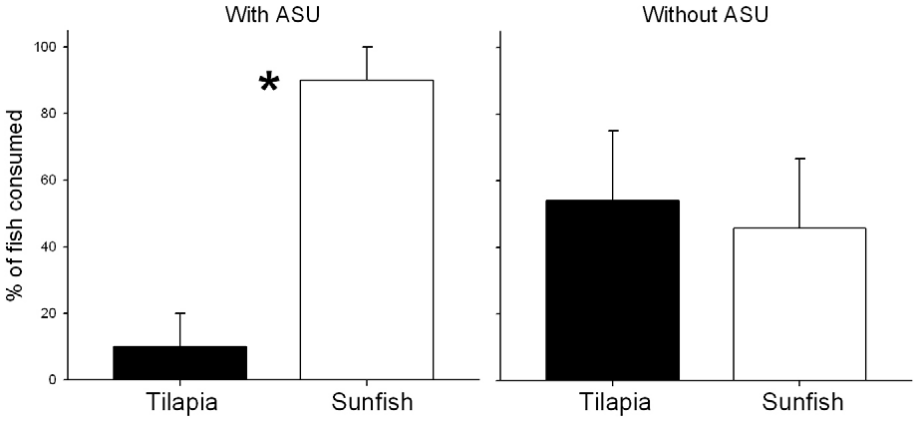
Consumption of tilapia and sunfish by largemouth bass (mean %±1 standard error) with (a) and without (b) artificial structure as a refuge. Asterisk indicates significant differences between species (p≤0.05).

In trials without structure, we found no evidence that bass preferred native sunfish over tilapia or vice versa (Figure 2; t(4) = −1.38, p = 0.262). However, when structure was present, largemouth bass consumed significantly more sunfish than tilapia (Figure 2; t(4) = −4, p = 0.016). Data satisfied assumptions of normality (without ASU: D = 0.304, p = 0.773; with ASU: D = 0.473, p = 0.151) and homogeneity of variance (without ASU: χ^2^ = 0.000, p = 0.996; with ASU: χ^2^ = 0.000, p = 1.000).

## Discussion

Recent reviews have suggested a need for scientists, conservationists, and environmentalists to examine the primacy of the historical paradigm that invasive species will reduce the biodiversity of natural ecosystems [4], [11]. These investigators showed that early predictions in the field of invasion ecology (i.e., principles such as competitive exclusion and native species extinction) are not always supported by the data [4], [9], [10], [70]–[72]. Comparisons of long term data frequently detected positive correlations between the distributional patterns of native and exotic plant species [e.g., [10]–[11]], suggesting that competitive exclusion of native species (sessile organisms and plants) by invasive species does not universally occur in lower trophic levels. Still, caution is needed when considering these examples as 1) many studies are focused on invasive plants [73], 2) certain areas have received little attention [i.e.], [ estuaries; 74–75], and 3) the tendency to publish positive results [76].

Among the most successful of the predictions made to date about invasive species is that invasive higher order, vagile consumers do have a great impact on native species, and in many cases led to their local extinction [77]. A recent analysis of long term extinction data shows that predation by invasive species is more likely to reduce the local native abundances than is competitive exclusion [11]. In particular, Sax and Gaines [11] note that over 80% of the vertebrate extinctions on islands were attributable to predation. The best documented examples include avifaunal extinctions on islands that have been attributed to increases in predation via mammal [78] and brown tree snake invasion [79]–[80].

Invasive fish are known to strongly impact native community structure in many ecosystems. Relevant examples include round gobies [81], common carp [82], salmonids [83], [84], and Nile perch [85], to name just a few. Our results show that the unintended release of the common aquaculture fish, Nile tilapia, can have negative impacts on the survival of native fishes in the oligohaline reaches of estuaries in the NGOM. Given that top down forces strongly influence most estuarine communities [17], we suggest these findings are applicable to a number of systems containing tilapia and perhaps other aggressive invasive cichlids. These impacts, however, are likely not limited to the competitive exclusion of native fishes from their preferred habitat. Tilapia may also prey on the eggs of many higher trophic level species, such as centrarchid fish, and adult tilapia may be more competitive with larger consumers all of which could further exacerbate their impacts on native ecosystems and food webs (although this is as yet undocumented in the scientific literature).

Since tilapia have been routinely recorded in the region [e.g., [58],[86]], it seems unlikely that the historical explanation of why tilapia do not represent a threat to native ecosystems is inaccurate (tilapia are reported intolerant to temperatures below 10°C [62]). Despite this, recent evidence suggests that low temperatures are unlikely to be a major impediment to the year-round survival of tilapia throughout the southern United States. Tilapia are known to actively seek warmer refuges to survive short term drops in temperature [59], [63]. Furthermore, increasing sea surface temperatures, a reported byproduct of global warming, have been observed throughout the NGOM [64]. Locally, an inspection of weather station data recorded in the upper reaches of Mobile Bay, AL indicates that there are relatively few days in winter when water temperatures fall below 10°C (in 2005–2008 a total of 6, 4, 15, and 10 days occurred, respectively (Mobile Bay National Estuary Program, http://www.mymobilebay.com/). These low temperatures are unlikely to occur uniformly throughout estuaries of the NGOM, however, (the same period further south at Dauphin Island, AL experienced 3, 8, 12, and 12 d when temperatures were <10°C) and these measurements were made in surface waters, with thermal refuges are probably found in deeper waters. Furthermore, the management paradigm that tilapia may not tolerate estuarine temperatures may not apply to all other strains of aquaculture fish.

Evidence for cold water tolerance in many strains of tilapia is lacking [60]. Lowe et al. [87] demonstrated that Nile tilapia survive well at temperatures of 15°C. Other studies have shown tilapia to be less tolerant, with 30% survival occurring at 10°C [88], although it was noted that temperature tolerance varied with fish size [89]. Other tilapia species, such as blue tilapia [*Oreochromis auratus*; 7°C; 90] and redbelly tilapia [*Tilapia zilli*; some survival at 6.5°C; 91] are known to tolerate colder temperatures than Nile tilapia.

Tilapia also tolerate the range of salinities that typically characterize the drowned river valley estuaries of the NGOM. Studies show that many cichlids, including Nile tilapia, can tolerate salinities reaching 25psu [60], [92]–[93]. Lowe et al. [87], however, found >60% of individuals in their experiments survived at 50 psu, approximately 90% survival at 40 psu, and breeding and growth to occur at 30 psu. Other tilapias have similar tolerance (i.e., blue tilapia (*O. auratus*) can reproduce in 19 psu and survive in waters of 54 psu [94]–[95], Florida red tilapia are routinely grown between 12–18 psu [91]–[93], and Mossambique tilapia (*Oreochromis mossambicus*) can reproduce at 49 psu and survive up to 64 psu [96]–[97]).

Consumer control, and the subsequent byproducts of the presence of predators (collectively termed “top down effects”), has been posited to exert a regulating effect on ecosystem structure and function [12]–[17]. Given that ecosystems respond strongly to higher order consumers, it is logical to predict that invasive predators will have the strongest impacts on coastal ecosystems. Indeed, Sax and Gaines [11] indicate that consumers are responsible for more native species extinction on islands than plant invaders. Evidence to date has supported this, with strong negative effects occurring as a result of other invading consumers [see 98 and references therein].

Based on this evidence, it seems clear that new precautionary management should be taken to reduce the unintended release of tilapia and other aquaculture species into coastal environments. The increased anthropogenic disturbances [6], together with the warmer winters in the area [64], suggests that the northern Gulf of Mexico coastal areas are very susceptible to tilapia invasion and persistence. Furthermore, tilapia are often grown in outdoor aquaculture facilities and northern gulf is at risk of natural disturbances such as hurricanes [58], [99]. While the use of aquaculture holds great promise for decreasing fishing pressure on wild fish stocks, studies of this nature are necessary to understand the potential impacts of invasive tilapia on native fish.

## Supporting Information

Video S1 Interactions between Nile tilapia and redspotted sunfish. Documented instances of aggression initiated by tilapia and resulting in the competitive exclusion of sunfish from the structured habitat in the first experiment.
(11.32 MB MOV)
